# Is Familiality Associated with Downward Occupation Drift in Schizophrenia?

**DOI:** 10.4306/pi.2008.5.3.168

**Published:** 2008-09-30

**Authors:** Triptish Bhatia, Satabdi Chakraborty, Pramod Thomas, Amina Naik, Sati Mazumdar, Vishwajit L Nimgaonkar, Smita N Deshpande

**Affiliations:** 1 Training Program for Psychiatric Genetics in India, Dr RML Hospital, New Delhi, India.; 2 Genetic Susceptibility in Schizophrenia, Dr RML Hospital, New Delhi, India.; 3 Department of Biostatistics, University of Pittsburgh, Pittsburgh, USA.; 4 Department of Psychiatry and Human Genetics, University of Pittsburgh, Pittsburgh, USA.

**Keywords:** Occupation drift, Schizophrenia, Familiality, Unemployment, Socioeconomic

## Abstract

**Objective:**

Downward occupational drift has been extensively investigated in schizophrenia. It is known that certain illness related factors, such as severity, affect drift, but the impact of familial factors has not been investigated.

**Methods:**

Occupation drift was studied among patients with schizophrenia/schizoaffective disorder (SZ/SZA)(n=523) and 130 affected sib pairs (ASPs). Drift was analyzed in relation to familiality as well as demographic and clinical variables. For comparison one proband (one of the affected siblings) from each ASP was selected. Occupation drift was measured in relation to the most responsible job held, and with regard to head of the household (HOH) occupation status.

**Results:**

There was no significant difference between single affected and ASP probands in terms of occupational drift from the most responsible job (drifted 39.2% and 38% respectively) and with regard to HOH's occupation (drifted 88% and 82.8% respectively). A significant part of the sample remained unemployed in both single affected and ASP samples. Thus, there was no significant impact of familiality on these variables. However, marital status, pattern of severity, age at onset, gender were found to be associated with downward occupation drift in single affected probands while the only significant factor in familial probands was pattern of severity of severity when measuring in terms of downward drift from most responsible job.

**Conclusion:**

Though there is occupation drift in schizophrenia, there is no detectable impact of familial factors. Employment is associated with severity of delete.

## Introduction

Classic epidemiological studies of schizophrenia have consistently found an inverse relationship between social class and schizophrenia.^1^,^2^ The relationship has been explained by two well publicized theories. The 'Social Causation hypothesis' postulates that stress experienced by individuals from low socioeconomic status is different qualitatively and quantitatively from those of high socioeconomic status classes; this results in onset or exacerbation of schizophrenia. The alternate, more accepted hypothesis is the downward drift hypothesis, which argues that the illness itself causes either the descent of the individual into a lower socio economic class or the inability of the individual to elevate himself or herself from the lower socioeconomic class. The second hypothesis has the greatest empirical support and is one of the cardinal features of schizophrenia.^3^

Downward drift has been extensibly documented in different countries over the past five decades. High rates of unemployment (up to 90%) have been reported in schizophrenia (SZ).^4^,^5^ Patients in different countries and cultures drift from employment to unemployment^6^ - Finland,^7^ Nigeria.^8^ The European Psychiatric Services: Inputs Linked to Outcome Domains and Needs (EPSILON) study conducted recently in five European countries reported 80% unemployment overall.^9^

Occupation is affected negatively in several ways- by not being able to complete education,^10^-^12^ social decline due to illness resulting in loss of competitive employment, unemployment or underemployment,^6^,^11^ hospitalizations and deterioration in interpersonal relations. Other predictors of occupational and social change have been reported e.g. gender, duration of illness, positive and negative symptoms.^13^ In our earlier study in a smaller sample, downward occupational drift was reported in India and marriage was found to be an important predictor of drift.^14^ Dohrenwend and Dohrenwend^1^ in their classic study envisioned a possible genetic causation of this downward drift. Whether drift has genetic or familial origin is still to be studied. Does the genetic sharing, as well as shared environment, lead to earlier age of onset among affected sibling pairs, and therefore, greater deterioration in occupational status? Schizophrenia is familial and its heritability is estimated at 80%.^15^ There is relatively high concordance rate among siblings and families face additional stress when more than one sibling is affected with the illness. Hence it is expected that the drift may be proportionately affected.

Studies measuring social drift have considered either employment before the onset of illness, or employment compared to heads of their households (HOH) as defining 'social class of origin'.^16^ The latter is said to be more accurate as it "gives a better picture of patients' environment" prior to their onset of illness and during its early stages, including both biological factors and social/familial experiences in childhood and early adulthood.^16^ These factors are implicated in the development, manifestations and trajectory of schizophrenia. The social class of origin cannot be caused by schizophrenia unlike current social class. It is manifested by being unable to work or to fulfill one's occupational potential.^16^

After a seminal World Health Organization (WHO) study,^17^ few Indian studies measured occupational performance after illness onset in schizophrenia. In a previous paper^14^ we found marked downward occupational drift in a smaller sample (n=588). Downward drift was observed in 51.8% of cases. In this study both families with one person affected and more than one person affected were combined for analysis. Current unemployment was considered for downward drift. In the present study we proposed to test familial nature of downward drift by comparing drift in singletons with those of affected sib pair (ASP) families in Indian schizophrenia population, using a larger sample than our prior study.

## Methods

### Sample

Participants affected with SZ or SZA were recruited from within Delhi and its surrounding areas. They were recruited from a range of facilities in order to ensure a representative sample. The families comprised of Government hospitals catering to middle and low socioeconomic groups and psychiatrists catering to higher income groups. Two types of families were recruited - single affected participants along with both parents and participants with one or more siblings affected with SZ or SZA (sibpairs). All participants were older than 18 years of age, and provided written informed consent.

A total of 745 patients were recruited. Exclusion criteria were: age below 18 years, free of substance dependence or any neurological or medical illness interfering with diagnosis. From the present study, we also excluded families in which, apart from siblings, parents or other first degree relatives were affected with schizophrenia, bipolar disorder, psychosis on family history using the Family Interview for Genetic Studies (FIGS; Maxwell, 1992), resulting in a total sample size of 653 single affected (n=523) and ASP (n=130) participants. The sample overlaps with our earlier report.^14^

### Diagnosis

Detailed interviews of the patients with their family members were carried out using the Diagnostic Interview for Genetic Studies (DIGS)^18^ that was translated into Hindi and validated (http://www-grb.nimh.nih.gov/gi.html).^19^ Additional information was obtained from relatives. Board certified psychiatrists established the consensus diagnoses based on all available information using Diagnostic and Statistical Manual of Mental Disorders, fourth edition (DSM-IV) criteria.

### Assessing occupation drift

Occupation of the proband and HOH (mostly fathers) was coded according to the coding format of the DIGS. Additional information was obtained from relatives. We coded for most responsible occupation (the highest status of employment which the participant attained in his/her life), the present job (occupation which the participant was currently engaged) and occupation of HOH. If HOH had retired, his/her job status was coded as per the highest job before retirement.

#### Occupation was Coded as Follows from the Diagnostic Interview for Genetic Studies

1. Managerial/Professional specialty occupations (executive, administrative, professionals, writers, artists, athletes, etc).
2. Technical/Sales and administrative support occupations (technician, sales occupation, clerical job, etc).
3. Service occupations (private house-hold occupation, protective service occupation, etc).
4. Farming/Forestry/Fishing/Precision production (farm operators, fishing occupation, mechanics, repairers, precision production occupation, etc).
5. Operators/Fabricators/Laborers (machine operators, assemblers, handlers, equipment cleaners, helpers, etc).
6. Full time student (see below).
7. House wife/Homemaker (see below).
8. Retired (see below).
9. Unemployed/Disabled.

##### Recoded categories

The self reported occupation of some individuals was changed on the basis of additional information from relatives (n=52 participants). These individuals were recoded regardless of family history as follows.

##### Students

Many patients reported their most responsible occupation as 'student' even if they were not studying regularly and not attending college/school programs. Their current age, length of time at a particular study level and static 'status' in academic work suggested that this was merely a label rather than a genuine occupation. Hence, the occupation of 'student' was changed to 'unemployed' if they remained at the same educational level (no progress in terms of passing) for more than five years and the participant was above 30 years of age. The parents reported that the progress was almost absent in terms of passing exams or any other achievement related to education, so they were kept in student category. Ninetytwo participants reported their present occupation as student. After recoding only seventy remained in this category.

##### Housewives

Many women stated their occupation "as housewife" or homemaker even when they were not doing any useful work. Depending on detailed information from family members and severity of illness and consensus discussions with board certified psychiatrists, the occupation of these cases was recoded as unemployed if the participants were not doing any useful work at home. Out of 81 housewives, 30 women were recoded as unemployed.

##### Retired

Since our sample comprised of relatively young participants there were no participants in this category. For retired parents' the last occupation was considered as the reference occupation.

#### Occupation Drift was Measured in Two Ways

1. Presence or absence of decline from the participant's own most responsible occupation to his/her present occupation.
2. Decline from HOH's occupation to participant's present occupation.

#### Participants were Divided into Three Groups Based on Occupation Drift

1. Presence of downward drift (present occupation status of the participant was lower than his/her most responsible or father's occupation status).
2. No drift (present occupation status same as his/her most responsible or father's occupation status).
3. Remained unemployed: Participant was never able to get employment or begin a new occupation. The participant could not complete his/her education or training required for any occupation. The illness might have started before any employment and he/she could never reach to a level when he could get employment.

### Data analysis

Participants were divided into two groups depending on whether they belonged to singleton families or ASP families. Descriptive statistics were used to measure drift/no drift categories in both types of families. Only one sibling from each ASP was randomly selected for analyses. Multinomial logistic regression was used to check association of drift with various demographic and clinical variables in all categories using the SPSS (version 14.0).

Based on prior publications, the independent variables included were age, age at onset, years of schooling, marital status, gender, diagnosis, current Global Assessment of Functions (GAF), past GAF, duration of illness, types of symptoms, longitudinal course, and pattern of severity.

## Results

### Demographic details

There were a total of 523 participants belonging to single affected families, 455 with SZ (87%) and 68 with SZA (13%), of whom 296 were male (56.6%) and 227 (43.4%) female (Table 1). Among 280 participants belonging to 130 families where two or more siblings were affected with schizophrenia, there were 15 families with more than two siblings affected, one with four siblings affected and one with five siblings affected. All affected siblings were included in the analysis. Among these 158 were male while 122 were female, 254 with SZ and 26 with SZA.

Mean age of single affected participants was 28.36±7.65 years (Mean±Standard deviation, SD) while those of ASPs was 34.47±9.78 years. Single affected participants' mean age at onset was 21.4±5.49 years and those of ASPs 23.78±6.69 years. The mean education of single affected participants was 11.48±3.65 school years while those of ASPs was 11.46±3.86 school years.

### Comparison between singleton and affected sib pair participants

Among singleton participants, 205 (39.2%) showed drift from their most responsible job, 213 (40.7%) remained in the same status and 105 (20.1%) remained unemployed. Comparing present job status with that of their HOH: 456 (88%) participants showed downward drift, 41 (7.9%) had no drift and 21 (4.1%) were better in job status compared to the most responsible job of their HOH (five records were missing)(Table 1). Occupational drift among the ASPs was calculated and for comparison with single affected only one sibling of the sib pair (n=130): was selected (38%) showed drift from most responsible job, 45.7% remained in the same job and 16.3% could not get any employment (Table 1).

There was no significant difference on downward occupation drift between families with only one child affected versus families with more than one affected child (ASP families)(Table 1). However, number of persons staying unemployed was lower in participants belonging to ASP families. Alcohol also has detrimental effect on occupation so frequencies of persons taking alcohol were calculated. Among ASP families, 31.9% of all male participants ever took alcohol in their lifetime, 9.2% of male participants took alcohol regularly for six months in their lifetime and 28.1% ever smoked.

### Factors associated with drift among families with only one affected participant

To examine a possible role of alcohol abuse in drift, the number of participants taking alcohol was determined. While 25.7% of all male participants had taken alcohol at some time in their life only 3.7% took alcohol daily for at least six months at any time of their life as described by DIGS. This suggests that alcohol intake may not have been a factor in occupation drift. Only 32.7% males ever smoked in their lifetime. On multinomial regression analysis (Table 2) participants affected with SZ had significantly more drift than SZA (χ^2^=0.009) when compared with their most responsible occupation. However SZA patients significantly improved more from their father's occupation than SZ (χ^2^=0.007). GAF (current or past) was positively correlated with severity of symptoms. So DIGS question in psychosis section about pattern of severity of illness was included for analysis. Between positively correlated age and age at onset latter was selected as the covariate. There was no positive correlation between the severity of illness and marital status so both were included in the analysis. Considering pattern of severity of illness, participants showing 'severe deterioration' declined more than participants with moderate or mild deterioration when compared to their own most responsible job (χ^2^<0.001). Drift was lesser among participants with later age of onset (χ^2^<0.001).

'Drift from most responsible job' was associated with marital status - not married drifted more than married and were unemployed (χ^2^=0.005). Compared to HOH's occupation drift was associated with gender, males declined more than females (χ^2^<0.001). In this analysis more the severity of illness more the drift (χ^2^<0.001) and finally lower age of onset was associated with drift and unemployment (χ^2^<0.001)(Table 2).

Table 3 displays drifts in different types of occupations. Downward occupation drift was reported to the greatest extent in technical occupations, least in farming occupations.

### Families with two or more affected children

On multinomial logistic regression analysis severity of illness was associated with unemployment both when compared with most responsible occupation (*p*<0.001, OR 2.386, CI 95% 1.715-3.31) and when with father's occupation (*p*<0.001, OR 4.131, CI 95% 2.499-6.82).

The distribution of ASP participants in different occupation categories (both present and most responsible occupation) along with their HOH's occupation is presented in Table 3. Drift was highest in technical (47.5%) occupations and least in farming (4%). Student category showed the lowest age at onset (19.6 years). Drift between younger and elder siblings (calculated separately) was also not significantly different from drift in single affected families.

### Comparison between affected siblings

There were 130 ASP families. Affected siblings were compared between themselves to see whether there was a difference of drift between elder and younger siblings; the youngest siblings were not included in these analyses if three siblings were affected. Among elder siblings 52 (40.6%) showed drift from their 'most responsible job' and 76 (59.4%) showed no drift. Among the affected youngest sibs 54 (40.9%) declined from their 'most responsible job' and 78 (59.1%) remained in the same level of job. Thus there was no significant difference in occupation drift among affected sibs when taken together. Tetrachoric correlation was calculated and there was no significant correlation between sibs on occupation drift.

## Discussion

The present study supported the social drift phenomenon in terms of occupation as reported in literature.^20^ Approximately 34% of the participants drifted to lower occupations, and an additional 20% could never find employment. This is much higher than the general unemployment rate in India (7.8%- CIA fact sheet, 2006) and testifies to the relatively high disability due to the disorder. The participants who were not employed before onset of illness were unlikely to get jobs later. In addition, 88% of patients drifted to lower occupation than their HOH's occupation. The rate of unemployment in schizophrenia in this sample seems lower than that reported in other countries. Many were employed in government jobs which are protected by law. The tolerance for lower occupational performance is possibly higher since there are no social security benefits in India. Hence patients may be retained in the system even if their productivity is low. They may be given less stressful and less responsible jobs.

Married participants showed lower decline than the unmarried patients- a phenomenon reported in the literature.^21^ Marriage is a positive outcome which holds the decline. Women with their own home, spouse and children have household responsibilities and support from family. Many of those who could not shoulder minimum household responsibilities ended up divorced. In India more persons affected with schizophrenia are married as compared with developed countries.^25^

The magnitude of drift does not appear to vary with the number of affected siblings in a family. Among ASPs, drift was observed in 38% of the sample identical to the single affected families. Among ASPs 16.3% were unemployed, less than in the families with one affected person (20%). Significantly more number of individuals drifted downward in moderate and severely ill groups than in the groups with mild severity.

There was no significant difference between elder and younger sibling with respect to age at onset. After the elder child falls ill the expectations and attention of parents may be focused on younger siblings, saving him from greater occupational drift. The family may also seek treatment earlier.^22^ Hambrecht^22^ reported that families with a history of schizophrenia spectrum disorders were more perceptive with respect to positive symptoms of schizophrenia (hallucinations and delusions) than those without a family history, but the opposite appeared to be true of cases with respect to less specific signs such as changes in affect, behavior and negative symptoms. Families with positive family history recognize need for help solely in response to early signs.^23^ There was no difference in drift shown by elder sibling versus younger sibling.^24^ Wickham^24^ found that employment in schizophrenia was not affected if more than one member is ill in a family. However they found that premorbid functioning was associated with familiality. Even unaffected siblings of persons with schizophrenia have poor academic functioning and deterioration in academic performance. This puts these single affected families at par with ASP families. Employment depends on many variables (cognitive abilities, education, age at onset, etc) than family history alone.

Persons working in technical jobs both in single affected and ASP families showed the greatest drift as technical jobs required higher cognitive functions. Farmers showed the least drift as possibly, farming in India is a group effort and the whole family works in the farms with the affected person. It is possible farming families looked after the patients. On the other hand 36% of laborers drifted downward, suggesting that the illness affects even jobs requiring less cognitive abilities. Alternatively, these figures may reflect difficulties in gaining employment during difficult economic times. Participants with most severe illness drifted the most, possibly because most of the ill person's time was spent in illness or treatment. Similarly, in their 13 year longitudinal study on Nigerian patients^7^ reported an unexpectedly high proportion of unemployed patients.

In conclusion the present study suggests that though there is occupation drift in schizophrenia, it does not change with increasing number of affected siblings.

## Figures and Tables

**TABLE 1:**
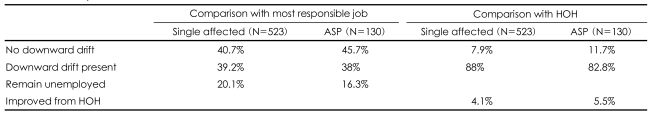
Drift in proband of single affected and affected sibpair families

ASP: affected sib pair, HOH: Head of Household

**TABLE 2:**
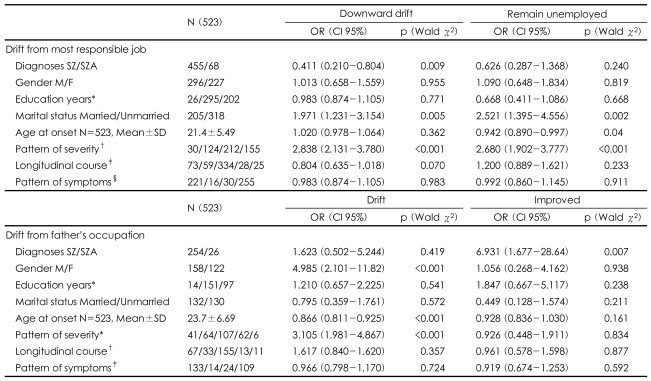
Odds ratios (confidence intervals) and p values of variables associated with downward occupational drift and unemployment compared to probands showing no drift in single affected families using multinomial regression analysis

^*^Coded education 1.0 to 5 yrs., 2.6-12 yrs. 3. above 12 yrs, ^†^Pattern of severity: 1. Episodic shift, 2. Mild deterioration, 3. Moderate deterioration, 4. Severe deterioration, ^‡^Longitudinal Course: 1. Episode with inter episodic residual symptoms, 2. Episodic, 3. Continuous, 4. Single episode in partial remission, 5. Single episode in full remission, ^§^Pattern of Symptoms: 1. Continuously positive, 2. Predominantly negative, 3. Positive converting to negative, 4. Mixture of positive and negative. SZ/SZA: schizophrenia/schizoaffective disorder, M/F: male/female

**TABLE 3:**
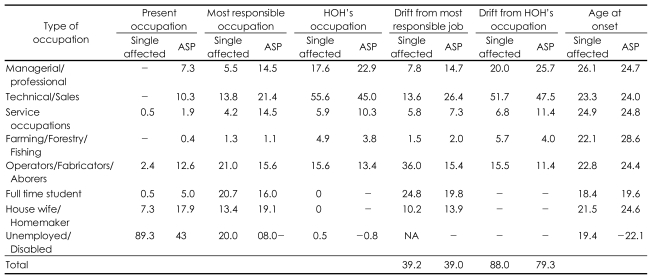
Percentage of downward drift in different occupations in probands of single affected and ASP families

ASP: affected sib pair, HOH: Phead of the household
